# *Mycobacterium tuberculosis* Uganda II is more susceptible to rifampicin and isoniazid compared to Beijing and Delhi/CAS families

**DOI:** 10.1186/s12879-016-1487-1

**Published:** 2016-04-21

**Authors:** George W. Kasule, David P. Kateete, Moses L. Joloba

**Affiliations:** National Tuberculosis and Leprosy Program, Kampala, Uganda; National TB Reference Laboratory, Kampala, Uganda; Department of Immunology and Molecular Biology, College of Health Sciences, Makerere University, Kampala, Uganda; Department of Medical Microbiology, College of Health Sciences, Makerere University, Kampala, Uganda

**Keywords:** *Mycobacterium tuberculosis* Uganda family, Minimum inhibitory concentrations, Drug susceptibility, Time-killing-curves, Isoniazid, Rifampicin, Strain, Genotype, Aerobic, Oxygen depleted

## Abstract

**Background:**

*Mycobacterium tuberculosis* Uganda family is the predominant cause of tuberculosis in Uganda. Reasons for this are not clear but are likely to be due to the rampant person-to-person transmission or delayed susceptibility of the organism to drugs during treatment, which may lead to continuous shedding of infectious bacilli, among others. The objective of this study was to determine in vitro, the susceptibility patterns of *M. tuberculosis* Uganda family compared with Beijing and Delhi/CAS, other *M. tuberculosis* sub-lineages that also circulate in Uganda but are not as prevalent. The comparisons were made after 10 days of exposure of the strains to Rifampicin and Isoniazid, the most important first-line anti-tuberculosis drugs.

**Methods:**

Minimum inhibitory concentrations (MICs) for three Isoniazid- and Rifampicin*-*susceptible *M. tuberculosis* strains (Uganda II, Beijing and Delhi/CAS families) were determined by micro-dilution plate assay. Killing curves for each strain were deduced from colony forming units after exposure to Isoniazid and Rifampicin on days 0, 2, 4, 6, 8, and 10 under aerobic and oxygen-depleted conditions. Data were analyzed with GraphPad Prism 5 software.

**Results:**

The MIC for Isoniazid was 0.05 μg/ml for *M. tuberculosis* Uganda II, and 0.03 μg/ml for *M. tuberculosis* Beijing and Delhi/CAS. Rifampicin MIC was 1 μg/ml for *M. tuberculosis* Uganda II, and 0.12 μg/ml for Beijing and Delhi/CAS. At low Rifampicin (0.03–2.5 μg/ml) and Isoniazid (0.12–5 μg/ml) concentrations under aerobic conditions, there was no significant difference in susceptibility patterns between *M. tuberculosis* Uganda II and Beijing or Delhi/CAS. However, at high Rifampicin (5 μg/ml) and Isoniazid (1.25 μg/ml) concentrations under oxygen-depleted conditions, *M. tuberculosis* Uganda II was more susceptible to the drugs compared with Beijing or Delhi/CAS families.

**Conclusion:**

The predominance of *M. tuberculosis* Uganda II family as the main causative agent of tuberculosis in Uganda is not attributed to its susceptibility behavior to Isoniazid and Rifampicin. Probably, its predominance is due to differences in the immune defenses in the general population against the strains, given that Beijing and Delhi/CAS families may have been introduced more recently. Further research beyond susceptibility to anti-tuberculosis drugs is required to fully explore tuberculosis strain predominance in Uganda.

## Background

Human tuberculosis is an important disease worldwide second only to HIV/AIDS as the greatest killer attributed to a sole infectious agent. Tuberculosis (TB) is caused by a group of closely related bacteria referred to as the “*Mycobacterium tuberculosis* complex” (MTBC) comprising *Mycobacterium tuberculosis, Mycobacterium bovis, Mycobacterium bovis* BCG*, Mycobacterium africanum, Mycobacterium canettii, Mycobacterium caprae, Mycobacterium microtii* and *Mycobacterium pinnipedii* [1, 2]*.* Although the MTBC are highly identical at DNA level, molecular genotyping data has revealed that genetic diversity in form of single nucleotide polymorphisms (SNPs), insertions & deletions (InDels), etc., occurs among the MTBC strains, and maybe responsible for phenotypic differences in drug susceptibility, pathogenicity, host tropism, transmissibility, immune response, and geographical distribution of the strains [1, 3]. In light of this, the MTBC are classified into seven lineages (Lineage 1 to 7) [1–3] globally distributed with some lineage-types being more predominant than others in certain geographical regions and human populations [2, 3]. Lineage 4 (Euro-American) is commonly found in Africa, America, and Europe while Lineage 2 (East-Asia) is prevalent in Asia [3]; all the seven human-adapted MTBC lineages do circulate in Africa where MTBC appears to have originated, accompanying human migrations over millennia [2].

*M. tuberculosis* Uganda is a sub-lineage of the *M. tuberculosis* lineage 4, the Euro- American lineage which is defined by RD 724 deletion [4–8]. The *M. tuberculosis* Uganda family comprises *M. tuberculosis* Uganda I and Uganda II subfamilies with the latter being more prevalent in Uganda [5, 7]. Asiimwe et al., 2008 [4], Nabyonga et al., 2011 [7], and more recently Ezati et al., 2014 [5], reported higher occurrence in Uganda of the *M. tuberculosis* Uganda subfamily (>70 %) compared with other genotypes combined (LAM, Delhi/CAS and Beijing families about 3 % each). However, little is known about the mechanisms that are responsible for this predominance in Uganda. Other than chance, one possibility is that the transmission of *M. tuberculosis* Uganda family in Ugandan populations is influenced by the level of drug susceptibility compared to the less predominant strains.

Furthermore, *M. tuberculosis* has a long generation time and capacity for dormancy, where its low metabolic activity complicates treatment options [9]. The bacilli can be located in pulmonary cavities, empyema, pus, or solid caseous material, where penetration by antibiotics is difficult or the pH is sufficiently low to inhibit the activity of most antibiotics [10]. Indeed, animal and human clinical trials have led to the concept that there are different populations of bacteria present within the host [11, 12]. Organisms in pulmonary cavities are thought to be multiplying in an aerobic environment and consequently behave in a way that can be mimicked by in vitro tests. Organisms located within caseous foci are in a milieu where the low pH is likely to inhibit the activity of agents such as aminoglycosides but provide the conditions necessary for pyrazinamide activity [13]. Bacteria found within macrophages probably exhibit occasional spurts of metabolism and may be in relatively microaerophilic conditions, where mycobacterial dormancy can be induced [13]. Organisms located within granulomas are in a milieu where the oxygen is depleted.

Most diagnostic susceptibility tests guiding TB treatment investigate metabolically active bacilli under static conditions and as such, they may not be informative with respect to time-kill kinetics of anti-TB drugs and the emergence of resistance in low metabolically active or even dormant mycobacterial cells [14]. The kinetics of antimicrobial activity is generally used to evaluate and compare new drugs and study differences and changes in the antimicrobial susceptibilities of clinically important bacterial isolates [15]. The time-killing-curve method has been used in many studies, since bacterial death is evaluated with more information by this method than by end-point methods. As a means of data analysis, mathematical modeling is anticipated to be a useful tool in describing and comparing kinetics, leading to quantitative appraisal of bactericidal effects [16]*.* The pharmacodynamics of TB treatment should be further explored, to prevent emergence of resistance, treatment failure, relapse of infection [14] and determine the drug response of different genotypes.

In this study, we used time-kill-kinetics of Rifampicin and Isoniazid, the most important first-line anti-TB drugs, against selected strains under aerobic and oxygen-depleted conditions, and determined the response of *M. tuberculosis* Uganda family to drugs, and whether this response might better explain its successful predominance in Uganda.

## Methods

### Study site and setting

This was a laboratory-based study conducted at the Uganda National Tuberculosis Reference Laboratory (NTRL) and the Medical & Molecular Laboratory of the Department of Medical Microbiology, College of Health Sciences, Makerere University. The NTRL is a fully equipped Biosafety Level 3 Laboratory where TB culture is routinely done. Molecular tests to confirm *Mycobacterium* isolates to species level, as well as strain families, were performed at the Medical & Molecular Laboratory.

### Isolates and drug susceptibility testing

*Mycobacterium tuberculosis* isolates were obtained from the Joint Clinical Research Centre (JCRC), Kampala. Drug susceptibility testing was previously performed at the JCRC and the isolates were found to be susceptible to both Isoniazid and Rifampicin. The isolates were previously confirmed to species level through Region of Difference PCR analysis [4], and genotyped by MIRU-VNTR to confirm they were *M. tuberculosis* Beijing, Uganda II, and Delhi/CAS families [7, 17].

At NTRL, the retrieved isolates (Beijing, Uganda II and CAS/Delhi) were sub-cultured by incubating at 37 °C for 21 days on Middlebrook 7H11 agar supplemented with OADC. Bacterial suspensions used in subsequent assays were prepared by resuspending single colonies of each strain into 1 ml of sterile distilled water.

Drug susceptibility testing was repeated using phenotypic and genotypic approaches; Middlebrook 7H10 agar proportion method and line probe assays (Genotype MTBDRplus, Hain Life Sciences, Germany), respectively. With both methods all the isolates were confirmed to be fully susceptible to Isoniazid and Rifampicin.

### Genotyping

Chromosomal DNA used as templates in PCRs was prepared as previously described [17]. Similarly, the *M. tuberculosis* spoligotypes were determined by Spoligotyping as recently described [6]. The isolates were re-genotyped using MIRU-VNTR [17] and SNP-based Real-Time PCR [8] to confirm that they belong to *M. tuberculosis* Beijing, Uganda II and Delhi/CAS families.

### Determination of minimum inhibitory concentrations

Isoniazid and Rifampicin were purchased from Sigma-Aldrich (St. Louis, MO). Stock concentrations at 10 mg/ml were prepared using either sterile distilled water (Rifampicin) or Dimethyl Sulphoxide (DMSO) (Isoniazid). To determine the minimum inhibitory concentrations (MICs), microdilution plate assays were performed as described by Wallace et al., 1986 [18] and Lenaerts et al., 2005 [19]. Bacterial suspensions were adjusted to McFarland standard of 0.5, 100 μl of which were inoculated into 11 ml Middlebrook 7H9 medium supplemented with glycerol and OADC, to give a desired inoculum of 1 x 10^5^ colony forming units (CFU) per ml.

Microtitre plate wells were seeded with 98 μl of the bacterial suspension, into which 2 μl of drug was added. The final concentration of drugs ranged from 0.03–4.0 μg/ml (Isoniazid) and 0.12-16 μg/ml (Rifampicin). Control wells contained only DMSO or water. The outer wells of the plates were filled with water to avoid evaporation from the sample wells. The plates were sealed with perforated parafilm and incubated at 37 °C for 21 days. Plates were observed weekly to monitor changes in growth. Growth inhibition was determined by visual examination. The MIC was determined as the lowest drug concentration with no visible bacterial growth after 3 weeks of incubation. Duplicate MICs per strain were determined. In all assays the *M. tuberculosis* reference strain, H37Rv, was used as the control.

### Determination of killing curves under aerobic conditions

The MICs above were used to calculate the final drug concentration per strain that was used in determining killing curves. The bacterial suspensions were adjusted to an inoculum of Log_10_ 7.0 CFU/ml (range of Log_10_ 6.9–8.2 CFU/ml), as described above. To the bacterial suspension, drugs were added to final concentrations of; 1x MIC, 2x MIC and 5x MIC, and incubated at 37 °C. As such, the concentrations for Isoniazid were: 0.5 μg/ml (1x MIC), 1 μg/ml (2x MIC) and 2.5 μg/ml (5x MIC) for Uganda II; 0.03 μg/ml (1x MIC), 0.06 μg/ml (2x MIC) and 0.15 μg/ml (5x MIC) for both Beijing and Delhi/CAS. Further, the concentrations for Rifampicin were: 1 μg/ml (1x MIC), 2 μg/ml (2x MIC) and 5 μg/ml (5x MIC) for Uganda II; 0.12 μg/ml (1x MIC), 0.24 μg/ml (2x MIC) and 0.6 μg/ml (5x MIC) for both Beijing and Delhi/CAS.

The day on which the drug was added to the cultures was defined as day 0 and the corresponding CFUs were taken as 100 %. On days 0, 2, 4, 6, 8 and 10, one milliliter was drawn from each tube and serially diluted with sterile distilled water. One hundred microliters of the diluted culture was plated onto Middlebrook 7H11 agar supplemented with OADC, and incubated at 37 °C under normal atmospheric conditions. After 21 days, CFUs/ml were determined and were used to calculate the log_10_ CFUs/ml.

### Determination of killing curves under oxygen depleted conditions

We employed Wayne’s model as described by Murugasu-Oei et al., 2000 [20] with a few modifications to suit our setting. Bacterial suspensions were prepared as explained above. Tubes were closed with sterile silicone rubber septa, and incubated at 37 °C with slow stirring for 24 days. Control tubes, in addition to the culture, contained the Methylene Blue dye (1.5 g/ml), which was used as an indicator of oxygen depletion. The blue dye faded and disappeared indicating that oxygen is successfully depleted in the culture, as described by Wayne and Hayes, 1996 [21]. The drugs were injected into cultures through the septa on day 24 at concentrations based on MICs. Controls had no drug but water or DMSO. The cultures were incubated at 37 °C for 10 days.

On days 0, 2, 4, 6, 8 and 10, one milliliter from each tube was serially diluted in sterile distilled water, and 100 μl of the diluted culture incubated at 37 °C on Middlebrook 7H11 agar plates supplemented with OADC under normal atmospheric conditions. Colonies were counted after 21 days.

### Data analysis

To test the hypothesis that there is a difference in response to anti-TB drugs between *M. tuberculosis* Uganda and other families, data were analyzed with the Student’s t-distribution test to compare responses of *M. tuberculosis* Uganda II vs. Beijing, and Uganda II vs. Delhi/CAS. The means of the different killing curves were compared and a *p*-value of <0.05 at 95 % CI and α = 0.05 was considered significant. The data was analyzed with GraphPad Prism statistical program version 5.

### Definitions

In this study, strain refers to any of the seven MTBC lineages [3] or sub-lineages; hence, strain = lineage/sub-lineage.

## Results

### Minimum inhibitory concentrations

The MIC for Isoniazid was 0.05 μg/ml for *M. tuberculosis* Uganda II, and 0.03 μg/ml for both Beijing and Delhi/CAS families. Rifampicin MIC was 1 μg/ml for *M. tuberculosis* Uganda II, and 0.12 μg/ml for both Beijing and Delhi/CAS. Thus, compared with the other two strains, *M. tuberculosis* Uganda II required higher Isoniazid and Rifampicin concentrations to inhibit its growth.

### Isoniazid is more effective under aerobic conditions than oxygen-depleted conditions

**Fig. 1:**
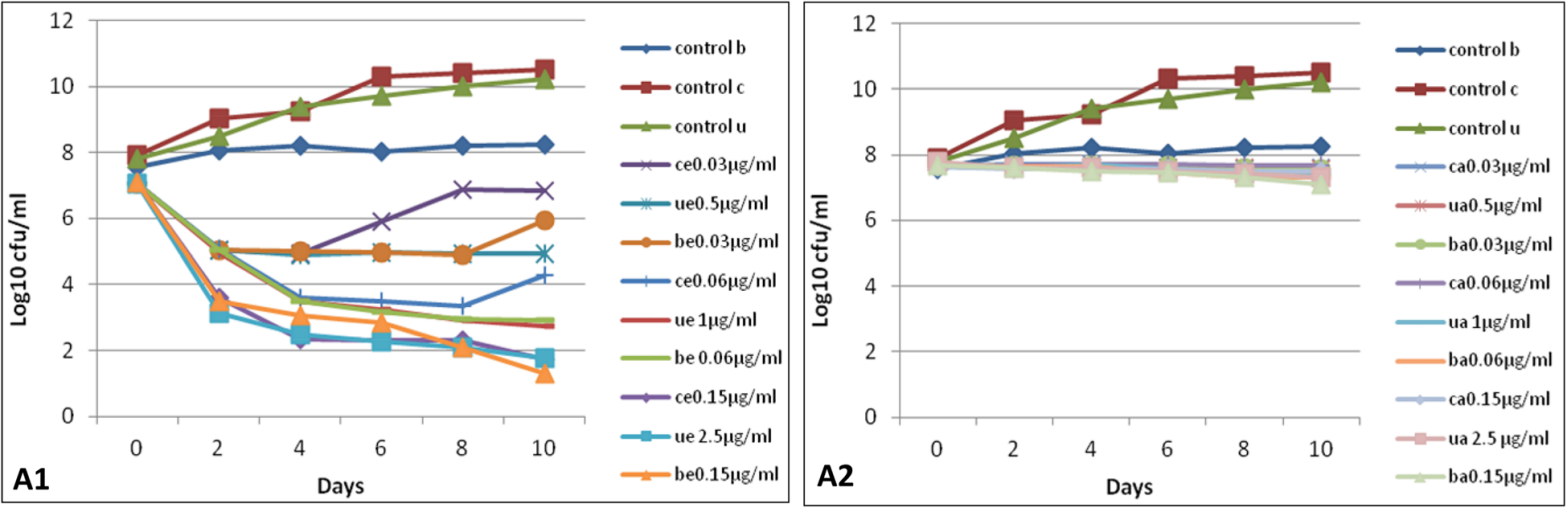
The effect of Isoniazid on *M. tuberculosis* Uganda II, Beijing and Delhi/CAS. *Panel A1*, aerobic conditions. control b, Beijing with no drug; control c, Delhi/CAS with no drug; control u, Uganda II with no drug; ce0.03 μg/ml, Delhi/CAS at 1x MIC; ue0.5 μg/ml, Uganda II at 1x MIC; be0.03 μg/ml, Beijing at 1x MIC; ce0.06 μg/ml, Delhi/CAS at 2x MIC; ue1 μg/ml, Uganda II at 2x MIC; be0.06 μg/ml, Beijing at 2x MIC; ce0.15 μg/ml, Delhi/CAS at 5x MIC; ue2.5 μg/ml, Uganda II at 5x MIC; be0.06 μg/ml, Beijing at 5x MIC. *Panel A2*, same as in *Panel A1* but under oxygen-depleted conditions

Under aerobic and oxygen depleted conditions, the CFU/ml of control cultures (no Isoniazid added) increased from days 0 to 10, Fig. 1. For instance, on day 0 the Log_10_ CFU/ml for *M. tuberculosis* Uganda II control was 7.8; it increased to 9.4 and 10.2 on days 4 and 10, respectively. On adding Isoniazid to cultures, CFUs/ml reduced both at low and high concentrations under aerobic conditions, Fig. 1. However, on adding Isoniazid under oxygen depleted conditions, CFUs/ml did not markedly reduce, Fig. 1. Hence, Isoniazid effectively killed more under aerobic conditions than under oxygen-depleted conditions. Table 1 shows the mean bacterial counts (Log_10_ CFU/ml) after 10 day exposure to Isoniazid and Rifampicin.

**Table 1:** Mean counts (Log_10_ CFU/ml) after 10-day exposure to Isoniazid and Rifampicin

Strain/ genotype	Isoniazid						Rifampicin					
	*Aerobic*			*Oxygen depleted*			*Aerobic*			*Oxygen depleted*		
	1x MIC	2x MIC	5x MIC	1x MIC	2x MIC	5x MIC	1x MIC	2x MIC	5x MIC	1x MIC	2x MIC	5x MIC
Uganda II	5.29	4.06	3.12	7.51	7.56	7.22*	5.45	4.11	2.86	5.03	3.91*	3.03*
Delhi/CAS	6.10	4.47	3.23	7.64	7.67	7.49	5.27	2.96	2.96	7.43	6.58*	5.23*
Beijing	5.48	4.10	3.30	7.64	7.51	7.36*	5.44	2.93	2.93	7.39	6.53	5.14*

*Where *P*-value was <0.05 when compared to Uganda II

Furthermore, under aerobic conditions comparison of the mean Log_10_ MICs of the pairs of killing curves for *M. tuberculosis* Uganda II vs. Beijing; *M. tuberculosis* Uganda II vs. Delhi/CAS was not statistically significant at all Isoniazid concentrations tested (*p*-value >0.05), Table 2. Likewise, under oxygen depleted conditions at low Isoniazid concentrations (1x MIC, 2x MIC), the differences in mean Log_10_ MICs of the pairs of killing curves for *M. tuberculosis* Uganda II vs. Beijing; *M. tuberculosis* Uganda II vs. Delhi/CAS was not statistically significant (*p*-value >0.05), Table 2. The exception was 5x MIC Isoniazid under oxygen depleted conditions, where we found the difference in mean Log_10_ MICs between *M. tuberculosis* Uganda II vs. Beijing to be statistically significant (*p*-value = 0.02, 95 % CI 0.019–0.169), Table 2.

**Table 2:** Analysis of pairs of killing curves for *M. tuberculosis* Uganda II vs. Beijing, and *M. tuberculosis* Uganda II vs. Delhi/CAS

		*1x MIC (Ug-0.5 μg/ml, Be-0.03 μg/ml, Ce-0.03 μg/ml)*	*2x MIC (Ug-1 μg/ml, Be-0.06 μg/ml, Ce-0.24 μg/ml)*	*5x MIC (Ug-0.25 μg/ml, Be-0.15 μg/ml, Ce-0.15 μg/ml)*
Drug (condition)	Strain	*p*-value, mean of diffs (95 % CI)	*p*-value, mean of diffs (95 % CI)	*p*-value, mean of diffs (95 % CI)
INH (aerobic)	Ug vs Be	0.30, −0.191 (−0.623–0.241)	0.40, −0.034 (−0.133–0.635)	0.33, −0.176 (−0.602–0.250)
	Ug vs Ce	0.09, 0.809 (−0.187–1.806)	0.14, 0.408 (−0.199–1.016)	0.30, 0.103 (− 0.128–0.334)
INH (O_2_ depletion)	Ug vs Be	0.13, −0.023 (−0.057–0.010)	0.17, 0.053 (−0.032–0.138)	0.02, 0.094 (0.019–0.169)*
	Ug vs Ce	0.20, 0.025 (−0.018–0.069)	0.14, 0.408 (−0.199–1.016)	0.55, 0.027 (−0.083–0.137)
		*1x MIC (Ug-1 μg/ml, Be-0.121 μg/ml, Ce-0.12 μg/ml)*	*2x MIC (Ug-2 μg/ml, Be-0.24 μg/ml, Ce-0.24 μg/ml)*	*5x MIC (Ug-5 μg/ml, Be-0.6 μg/ml, Ce-0.6 μg/ml)*
RIF (aerobic)	Ug vs Be	0.93, 0.006 (−0.162–0.174)	0.12, −0.427 (1.025–0.169)	0.60, −0.603 (−0.603–0.391)
	Ug vs Ce	0.34, −0.176 (−0.617–0.263)	0.07, 0.485 (−0.081–1.052)	0.49, 0.140 (−0.348–0.629)
RIF (O_2_ depletion)	Ug vs Be	0.51, −0.040 (−0.187–0.107)	0.36, 0.011 (−0.018–0.041)	0.004, −2.137 (−3.277–0.996)*
	Ug vs Ce	0.13, 0.073 (−0.033–0.179)	0.01, 0.036 (0.009–0.063)*	0.005, 2.230 (0.997–3.463)*

MIC, minimum inhibitory concentration; Ug, Uganda II; Ce, Delhi/CAS; Be, Beijing

INH, Isoniazid; RIF, Rifampicin;

*P-value <0.05

### Rifampicin is more effective than Isoniazid at killing *M. tuberculosis* under oxygen-depleted conditions

**Fig. 2:**
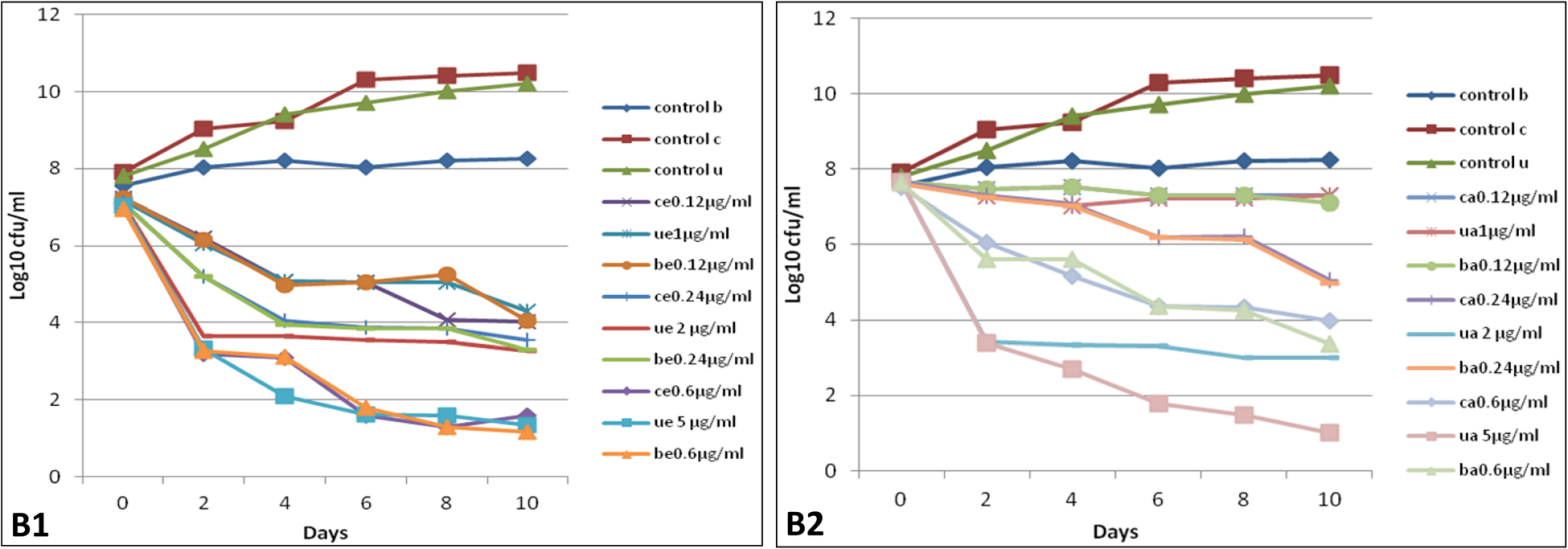
The effect of Rifampicin on *M. tuberculosis* Uganda II, Beijing and Delhi/CAS. *Panel B1*, aerobic conditions. control b, Beijing with no drug; control c, Delhi/CAS with no drug; control u, Uganda II with no drug; ce0.12 μg/ml, Delhi/CAS at 1x MIC; ue1 μg/ml, Uganda II at 1x MIC; be0.12 μg/ml, Beijing at 1x MIC; ce0.24 μg/ml, Delhi/CAS at 2x MIC; ue2 μg/ml, Uganda II at 2x MIC; be0.24 μg/ml, Beijing at 2x MIC; ce0.6 μg/ml, Delhi/CAS at 5x MIC; ue5 μg/ml, Uganda II at 5x MIC; be0.6 μg/ml, Beijing at 5x MIC. *Panel B2*, same as in *Panel B1* but under oxygen-depleted conditions

Similar to Isoniazid, the CFU/ml of controls where no Rifampicin was added, increased from days 0 to 10 both under aerobic and oxygen depleted conditions, Fig. 2. However, in contrast to Isoniazid, Rifampicin reduced the CFUs beyond day 0 under both aerobic and oxygen depleted conditions, Fig. 2. This result was more pronounced at high Rifampicin concentrations (2x MIC, 5x MIC) under oxygen depleted conditions, Fig. 2. Overall, both drugs were more effective at killing the bacilli under aerobic conditions compared to oxygen depleted conditions, although this difference was more pronounced for Isoniazid. Thus, compared to Isoniazid, Rifampicin had more killing ability of *M. tuberculosis* irrespective of strain or genotype under oxygen depleted conditions.

Similarly for Rifampicin at low concentrations under aerobic conditions, analysis of the pairs of the killing curves for *M. tuberculosis* Uganda II vs. Beijing; *M. tuberculosis* Uganda II vs. Delhi/CAS did not show significant difference in the means (*p*-value >0.05), Table 2. Also, under oxygen depleted conditions at 1x MIC Rifampicin concentration there was no significant difference in the killing curves (*p*-value >0.05). However, in contrast to Isoniazid, Rifampicin showed more pairs under oxygen depleted conditions, with significant differences in mean MICs even at 2x MIC; *M. tuberculosis* Uganda II vs. Delhi/CAS (*p*-value = 0.01, 95 % CI 0.009–0.063 at 2x MIC; *p*-value = 0.005, 95 % CI 0.997–3.463 at 5x MIC); *M. tuberculosis* Uganda II vs. Beijing (*p*-value = 0.004, 95 % CI −3.277–0.966) at 5x MIC, Table 2.

Taken together, these data show that *M. tuberculosis* Uganda II is more susceptible to Isoniazid and Rifampicin than Beijing or Delhi/CAS families, particularly at high drug concentrations under oxygen depletion.

## Discussion

We have investigated drug-resistance and virulence as factors associated with the predominance of the *M. tuberculosis* Uganda family in Uganda. In one of the studies, we found that the *M. tuberculosis* Uganda family is three times less likely to be resistant to first-line anti-TB drugs and five times less likely to be multidrug resistant, compared to the non-Uganda II families [5]. Furthermore, in the other study investigating virulence we found that the *M. tuberculosis* Uganda family was less virulent as defined by cavitary disease [8]. Although response to first-line anti-TB drugs would offer a better explanation to predominance, in this study, we have found no significant differences in the susceptibility patterns between *M. tuberculosis* Uganda II vs. Beijing or Delhi/CAS families, except at high drug concentrations under oxygen-depleted conditions where the Uganda II family was instead more susceptible to Rifampicin and Isoniazid than the Beijing or Delhi/CAS families. Therefore, in line with recent studies [4–6], the predominance of *M. tuberculosis* Uganda family in Uganda may not be attributed to its susceptibility behavior once exposed to Isoniazid and Rifampicin.

The MICs for Rifampicin and Isoniazid showed that the strains were fully susceptible to both drugs and the killing curve phenomenon could be demonstrated. The MICs for Isoniazid were in agreement with previous reports [22] of 0.02–0.05 mg/ml and were equally effective against all the three MTBC strains. Also, the MICs for Rifampicin were in agreement with the previous study [23] that reported 0.25–0.5 mg/L and 1 mg/L using agar proportion method and micro-dilution resazurin assay, respectively, for *M. tuberculosis* laboratory strain H37Rv commonly used as the reference for in vitro studies, and 0.0625–0.125 mg/L for susceptible clinical isolates.

Generally under oxygen-depleted conditions, the less predominant strains (*M. tuberculosis* Beijing and Delhi/CAS families) were less susceptible at the highest test concentrations of Isoniazid and Rifampicin (1.25 μg/ml and 5 μg/ml, respectively). These strains had lower MICs than *M. tuberculosis* Uganda II and probably respond better in vivo where drug concentrations are higher than the ones studied. Nevertheless, our test concentrations are still lower than the peak concentrations of both drugs in serum (3–7 μg/mL and 6–9 μg/mL for isoniazid and rifampicin, respectively) [24], implying that different responses may occur in vivo.

Although Isoniazid showed high and rapid activity against replicating mycobacteria under aerobic conditions, compared to Rifampicin, it exhibited poor activity against them under conditions of low oxygen tension. Increasing the drug concentrations did not change this observation. This could be attributed to the fact that Isoniazid targets *Mycobacterium*’s cell wall synthesis [19] and is therefore more effective against actively replicating bacteria. The rapid activity of Isoniazid is further supported by similar findings of de Steenwinkel et al., 2010, where it also showed extremely rapid and concentration-dependent killing while Rifampicin had relatively slow and strongly time-dependent killing [14]. Furthermore, at low drug concentrations we noted no significant difference in the way the three strains responded to Isoniazid and Rifampicin under aerobic conditions. This agrees with findings by Lenaert et al., 2005, where Isoniazid showed little activity against the non-replicating bacteria even when 2 μg/ml, 10 μg/ml, and 50 μg/ml concentrations were used [19]. On the other hand, Rifampicin showed a better activity than Isoniazid at all concentrations tested under conditions of low oxygen tension. This is supported by Lenaert et al., 2005 findings*,* where 2 μg/ml, 10 μg/ml and 50 μg/ml of Rifampicin was very effective against non-replicating *M. tuberculosis* grown under conditions of oxygen depletion [19]. This is because Rifampicin targets protein synthesis and is therefore active against non-replicating bacteria or low metabolizing bacteria [19] where it inhibits RNA synthesis by inhibiting DNA-dependent RNA polymerase [25].

The inactive state of the tubercle bacilli was achieved as described by Wayne and Hayes, 1996, resulting in a state known as non-replicating phase 2 (NRP-2) of low metabolic rate that is experienced during the conditions of low oxygen tension [21]. However, apart from using Methylene Blue dye to indicate that the low oxygen level condition is achieved, we could not accurately measure the level of oxygen tension [21].

In Uganda, latent and active TB are treated with similar drug regimen irrespective of the strain causing disease in the host. Given our findings, one can infer that there is no need of genotyping the infecting strain of a patient before starting treatment or when judging prophylaxis, since there is no significant difference in killing. However, further research into this is required to enable detailed comparisons; testing more strains at wider range of drug concentrations that put into consideration the serum levels achieved during treatment is recommended.

## Conclusions

At low Rifampicin and Isoniazid concentrations under aerobic conditions, there is no difference in susceptibility patterns between *M. tuberculosis* Uganda II and Beijing or Delhi/CAS families. At high concentrations of the same drugs under oxygen-depleted conditions, *M. tuberculosis* Uganda II is more susceptible to Rifampicin and Isoniazid compared to Beijing or Delhi/CAS families. Therefore, the predominance of the *M. tuberculosis* Uganda II family as the main causative agent of TB in Uganda cannot be attributed to its susceptibility behavior as judged from the three selected strains. Probably, its predominance is due to differences in the immune defenses in the general population against the strains, given that the *M. tuberculosis* Beijing and Delhi/CAS families are likely to have been introduced more recently. Further research beyond susceptibility to anti-TB drugs is required to ascertain these.

### Ethics statement

This study was nested within previously approved studies [8, 17] that obtained approval from the Joint Clinical Research Centre Kampala, the University Hospitals Cleveland Institutional Review Board (Cleveland Ohio), and Makerere University School of Medicine Institutional Review Boards. Informed written consent (including the use of samples in further studies) was obtained from patients who participated in the study. The study was also reviewed and approved by the Higher Degrees Research Committee of the College of Veterinary Medicine, Animal Resources and Biosecurity, Makerere University. The *M. tuberculosis* test isolates in this study were originally cultured from collected human samples.

### Availability of data and materials

The datasets supporting the conclusions of this article are included within the article.

## Abbreviations

CI: confidence interval
JCRC: joint clinical research center
MIC: minimum inhibitory concentration
MRA: micro-dilution resazurin assay
NRP-2: non-replicating phase 2
NTRL: national tuberculosis reference Laboratory
OADC: Oleic Albumin Dextrose Catalase
PCR: polymerase chain reaction
